# An Advanced Numerical Approach to Evaluate the Maximum Bonding Force of Rock Anchors

**DOI:** 10.3390/ma19204395

**Published:** 2026-10-19

**Authors:** Jianhang Chen, Jie Fang, Yong Zhang, Chenyang Zhu, Krzysztof Skrzypkowski, Krzysztof Zagórski, Anna Zagórska, Peng Du

**Affiliations:** 1 State Key Laboratory of Water Resource Protection and Utilization in Coal Mining, Beijing 102211, China; 2 School of Energy and Mining Engineering, China University of Mining and Technology (Beijing), Beijing 100083, China; 3 Faculty of Civil Engineering and Resource Management, AGH University of Krakow, Mickiewicza 30 Av., 30-059 Krakow, Poland; 4 Faculty of Mechanical Engineering and Robotics, AGH University of Krakow, Mickiewicza 30 Av., 30-059 Krakow, Poland; 5 Research Centre in Kraków, Institute of Geological Sciences, Polish Academy of Science, Senacka 1, 31-002 Krakow, Poland; 6 Huafeng Coal Mine of Xinwen Mining Group Co., Ltd., Taian 271413, China

**Keywords:** rock supporting, anchorage mechanism, stress transfer, roof stability, grout shear strength

## Abstract

To quantitatively evaluate the maximum bonding force (BF) of rock anchors, this paper conducts an analytical investigation and numerical calculation. An advanced numerical approach is proposed. During the tensioning process of rock anchors, in the elastic-softening–debonding course, the debonding region is continuously divided into five different points. Then, for these individual points, the corresponding BF is calculated. Computational loops are created to conduct the calculation until the maximum BF is obtained. Two individual experimental anchorage tests on long-grouted rock anchors were adopted to validate the effectiveness of this current approach. A comparison was conducted between the traditional and current approach. The results showed that the anchorage performance obtained with the current approach agreed well with the traditional approach. However, compared with the traditional approach, the computational efficiency of the current approach is significantly improved. The superiority of this current approach is independently reflected by the number of elements in the softening region. The grout shear strength has a significantly beneficial influence on improving the maximum BF. This study is beneficial for quantitatively evaluating the maximum BF of rock anchors with high efficiency.

## 1. Introduction

**Figure 1:**
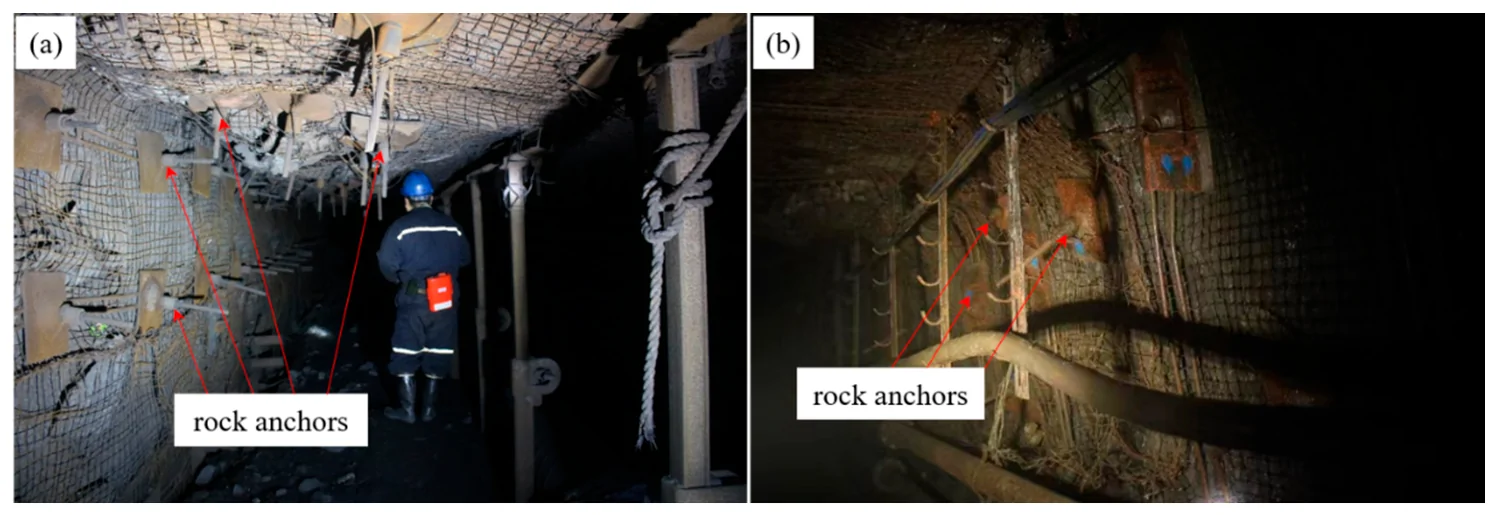
Installing rock anchors to prevent collapsing of rock masses around underground excavations: (**a**) rock anchors are used with steel mesh; (**b**) rock anchors are installed at the lateral side of roadways.

Rock anchors are widely used in mining and civil construction [1]. Generally, rock anchors should be used with grouting materials to realise their function. Currently, two different grouting materials are commonly used. One is resin grout and the other one is cement-based grout [2]. In practical cases, operators can drill boreholes in rock masses around underground excavations. Then, rock anchors can be inserted into these drilled boreholes. To establish the connection between rock anchors and rock masses around underground excavations, resin or cement-based grout can be pumped into the annulus between rock anchors and rock masses. Once the resin or cement-based grout cures, it can bind rock anchors and rock masses together. Then, rock anchors, grout and rock masses can form an integrity. This is effective in improving the overall strength and stability of rock masses around underground excavations (Figure 1).

After rock anchors are installed, engineers and researchers pay more attention to the maximum bonding force (BF) of rock anchors. This is because before rock anchors reach the maximum BF, rock anchors can continuously provide deformation resistance for rock masses around underground excavations. Once rock anchors reach the maximum BF, the rock bolting system enters the post-failure state. Then, the BF drops quickly. Once large displacement generates between rock anchors and rock masses around underground excavations, rock anchors are likely to provide less deformation resistance. Therefore, the maximum BF is a significant factor that determines the stability of rock masses around underground excavations.

To evaluate and predict the maximum BF, researchers and engineers always adopt the analytical modelling method [3]. Compared with experimental anchorage tests on rock anchors, an advantage of analytical modelling is that users can establish the mechanical relationship between rock anchors, grout and rock masses. Then, based on the established analytical model, users can quantitatively calculate the mechanical performance under different geotechnical environments.

To evaluate the maximum BF, Aydan, et al. [4] indicated that a tri-linear curve was effective in simulating the elastoplastic behaviour of the interface in rock reinforcement systems. Moreover, based on this concept, they obtained the maximum BF. Later, this model was adopted and extended by various researchers [5,6,7,8,9,10]. Moreover, this model was incorporated into numerical simulation programmes to predict the maximum BF [10].

Yazici and Kaiser [11] conducted theoretical analysis to evaluate the maximum BF. The bond effect of the interface between rock anchors and grout was especially analysed. They found that increasing the grout stiffness can improve the maximum BF. Hyett, et al. [12] estimated the BF through developing a constitutive model to estimate the BF. Basically, a satisfactory agreement between the modelling and experimental results occurred. Moosavi [13] referred to the continuous yielding model and extended it to calculate the BF. They found that the maximum BF was sensitively influenced by the effective shear stiffness of grout. Cao, et al. [14] focused on the rock anchors’ profile. They revealed the relationship between the maximum BF and rib face angles. Ghadimi, et al. [15] also concentrated on the influence of the rock anchor profile on the BF. They found that the maximum BF was critically determined by the rib height and spacing. Ali, et al. [16] analysed the anchorage performance based on artificial neutral networks. They found that the curing time of grout and the mechanical properties of rock masses apparently influenced the maximum BF. Li, et al. [17] analysed the mechanical interlock between rock anchors and grout. They developed a non-linear equation to calculate the anchorage performance. An advantage of their model was that the post-failure performance can be successfully captured. Therefore, the maximum BF can be properly calculated. Liang, et al. [18] paid attention to the cone structure created between rock anchors and grout. For rock anchors, they created a conceptual model through considering the anchorage function of the cone structure. They found that partial shear failure around the cone structure led to a decrease in the maximum BF. Li, et al. [19] adopted analytical modelling to simulate the anchorage performance under constant normal stiffness conditions. The results showed that their analytical model can successfully capture the maximum BF of two types of rock anchors.

Among different kinds of analytical models, the tri-linear curve is commonly used by various researchers [9,20]. The tri-linear curve can be calculated with Equations (1)–(3) [7]:

(1) $$\tau =\frac{\tau_{p}}{\delta_{p}}\delta_{s}\left(0\leq \delta_{s}<\delta_{p}\right)$$

(2) $$\tau =\frac{\tau_{p}\delta_{r}-\tau_{r}\delta_{p}}{\delta_{r}-\delta_{p}}-\frac{\tau_{p}-\tau_{r}}{\delta_{r}-\delta_{p}}\delta_{s}\left(\delta_{p}\leq \delta_{s}<\delta_{r}\right)$$

*τ* = *τ_r_*(*δ_s_* ≥ *δ_r_*) (3)

where *τ*: the shear stress at the anchor/grout interface; *τ_p_:* the shear strength; *δ_p_*: the shear slip at the shear strength; *δ_s_*: the shear slip at the anchor/grout interface; *δ_r_*: the shear slip at the residual shear strength; and *τ_r_*: the residual shear strength.

In this study, it was assumed that the grout shear strength equalled the shear strength of the anchor/grout interface. In fact, the shear strength of the anchor/grout interface was more complicated. It was closely influenced by the properties of the rock anchors and grout. Compared with that, the grout shear strength can be easily measured by direct shear tests or tri-axial tests. Therefore, for simplification, this study used the grout shear strength to represent the shear strength of the anchor/grout interface.

**Figure 2:**
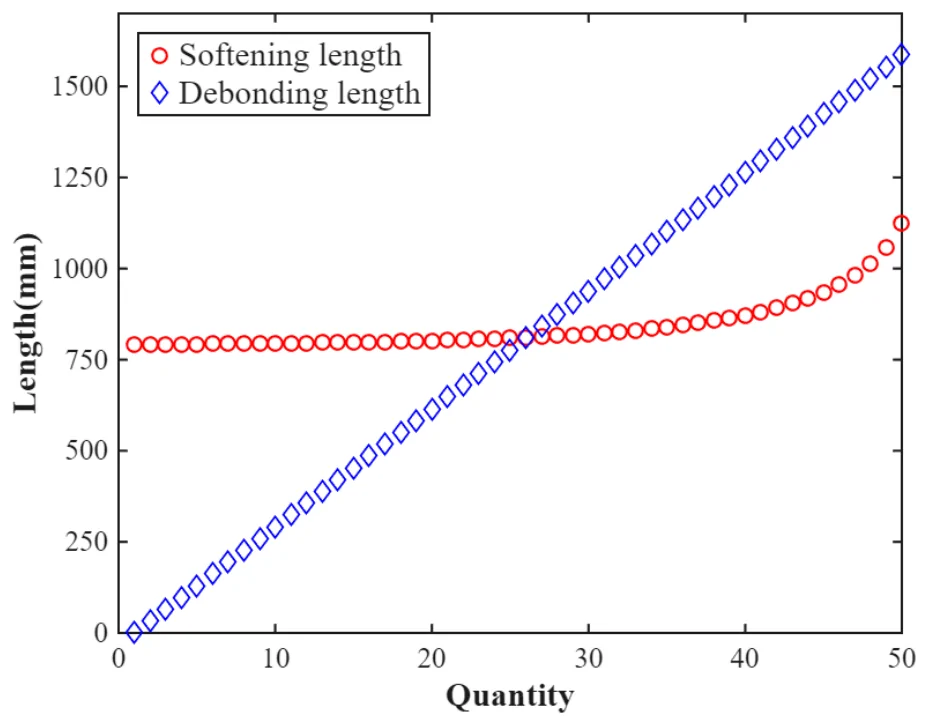
Typical variation process of the softening length and debonding length when calculating the maximum BF.

The tri-linear curve considers the elastoplastic behaviour of the anchor/grout interface. Therefore, it can simulate the pre-failure and post-failure of the interface. However, when the tri-linear curve is used, a shortcoming still exists. To calculate the maximum BF, along the whole length, the softening region and debonding region should be determined. However, during the tensioning process, these two lengths vary simultaneously (Figure 2).

To calculate the proper softening length and debonding length, the existing algorithm requires that the debonding length can be increased from zero to its maximum value. During this increasing process, a small interval can be added to the initial debonding length. Then, this small interval can be added cyclically to gradually increase the debonding length. Meantime, the calculation will be conducted to capture the maximum BF. However, this requires a number of iteration counts and its computational efficiency is low.

To simplify the calculation process, one method is to assume that the softening region remains unchangeable [21]. However, this cannot truly reflect the exact reinforcement scenario where the softening region increased continuously before reaching the maximum BF.

To overcome this shortcoming, for the tri-linear curve, this paper proposed an advanced numerical approach to evaluate the maximum BF. This paper is written with the following structure. First, the fundamental principle of this current approach is illustrated. Two flow charts are drawn to explain the computational structure of this current approach. Second, two experimental anchorage tests are adopted to validate the accuracy of this current approach. Moreover, the comparison is conducted between the traditional and current approach, confirming the superiority of this current approach. Finally, based on this current approach, parameter studies are conducted to evaluate its computational efficiency. Based on this current approach, the influence of the grout shear strength and grout shear stiffness on the maximum BF is studied.

## 2. Fundamental Principle of the Current Approach

### 2.1. Overall Computational Structure of the Current Approach

In this study, the tri-linear curve shown in Equations (1)–(3) is used to depict the slip behaviour of the anchor/grout interface. To realise the calculation, it is assumed that after the rock anchoring system is loaded, the grout and rock deform elastically. This assumption is based on the following fact. Previous experimental anchorage tests showed that after the rock anchoring system was loaded, the anchor/grout interface slip is the dominant failure mode [9]. As for the grout and rock, they are still in the elastic state.

When the tri-linear curve is incorporated into rock anchoring systems, for rock anchors, it is believed that their tensioning process can be divided into several courses. They are the initial elastic course, the second elastic-softening course, the third elastic-softening–debonding course, the fourth softening–debonding course and the final debonding course [9].

Previous research demonstrated that the maximum BF usually occurred in the third course [8]. This is because when the interface starts debonding, the shear stress along the elastic region is still increasing. Meantime, for the softening region, its length increases. Then, the stress along the softening region is increasing. Therefore, under the mutual influence of the elastic region and softening region, the BF continuously increases. Once the debonding length increases to its critical value, the BF reaches the maximum.

In the third course, the whole interface can be separated into three regions: the elastic region, softening region and debonding region.

In the third course, the BF can be calculated with Equation (4) [22]:

(4) $$F=2\pi r_{b}\left(\frac{\tau_{p}}{\lambda_{1}}\tanh\left(\lambda_{1}\left(L-a_{d}-a_{s}\right)\right)\cos\left(\lambda_{2}a_{s}\right)+\frac{\tau_{p}}{\lambda_{2}}\sin\left(\lambda_{2}a_{s}\right)+\tau_{r}a_{d}\right)$$

where *F*: the BF; *r_b_*: the rock anchors’ radius; *λ*_1_: an elastic coefficient, which can be calculated with Equation (5); *L*: the anchorage length; *a_d_*: the debonding region’s length; *a_s_*: the softening region’s length; and *λ*_2_: a plastic coefficient, which can be calculated with Equation (6).

(5) $$\lambda_{1}^{2}=\frac{\tau_{p}}{\delta_{p}}\lambda^{2}$$

(6) $$\lambda_{2}^{2}=\frac{\tau_{p}-\tau_{r}}{\delta_{r}-\delta_{p}}\lambda^{2}$$

where *λ*: a common coefficient used for the elastoplastic calculation, which can be calculated with Equation (7).

(7) $$\lambda^{2}=\frac{2}{r_{b}}\left(\frac{1}{E_{b}}+\frac{\pi r_{b}^{2}}{E_{m}A_{m}}\right)$$

where *E_b_*: the rock anchor modulus; *E_m_*: the surrounding rock modulus; and *A_m_*: the cross-sectional area of surrounding rock.

To obtain the critical length of the softening region and debonding region when reaching the maximum BF, the iterative calculation is always adopted. The traditional approach follows the below procedures. The debonding length can be increased from zero to its final value in the third course, which can be calculated with Equation (8):

*a_df_* = *L* − *a_sf_* (8)

where *a_df_*: the debonding region’s final length in the third course; and *a_sf_*: the softening region’s final length in the third course, which can be calculated with Equation (9).

(9) $$a_{\mathrm{sf}}=\frac{1}{\lambda_{2}}\arccos\left(\frac{\tau_{r}}{\tau_{p}}\right)$$

For a specific debonding length, the softening length can be increased from its initial value in the third course (*a_si_*) to its final value in the third course (*a_sf_*). As for the initial length of the softening region in the third course, it can be calculated with Equation (10).

(10) $$a_{\mathrm{si}}=\frac{1}{\lambda_{2}}\left(\arcsin\frac{\lambda_{1}}{\sqrt{\lambda_{1}^{2}+\lambda_{2}^{2}}}-\arcsin\frac{\tau_{r}\lambda_{1}}{\tau_{p}\sqrt{\lambda_{1}^{2}+\lambda_{2}^{2}}}\right)$$

where *a_si_*: the initial length of the softening region in the third course.

Based on this calculation, the proper length of the softening region can be obtained. For each pair of softening regions and debonding regions, the BF can be obtained. After obtaining all BFs in the third course, the maximum BF can be obtained. However, the above analysis requires a vast number of iteration counts. This reduces the computational efficiency of the iterative calculation.

**Figure 3:**
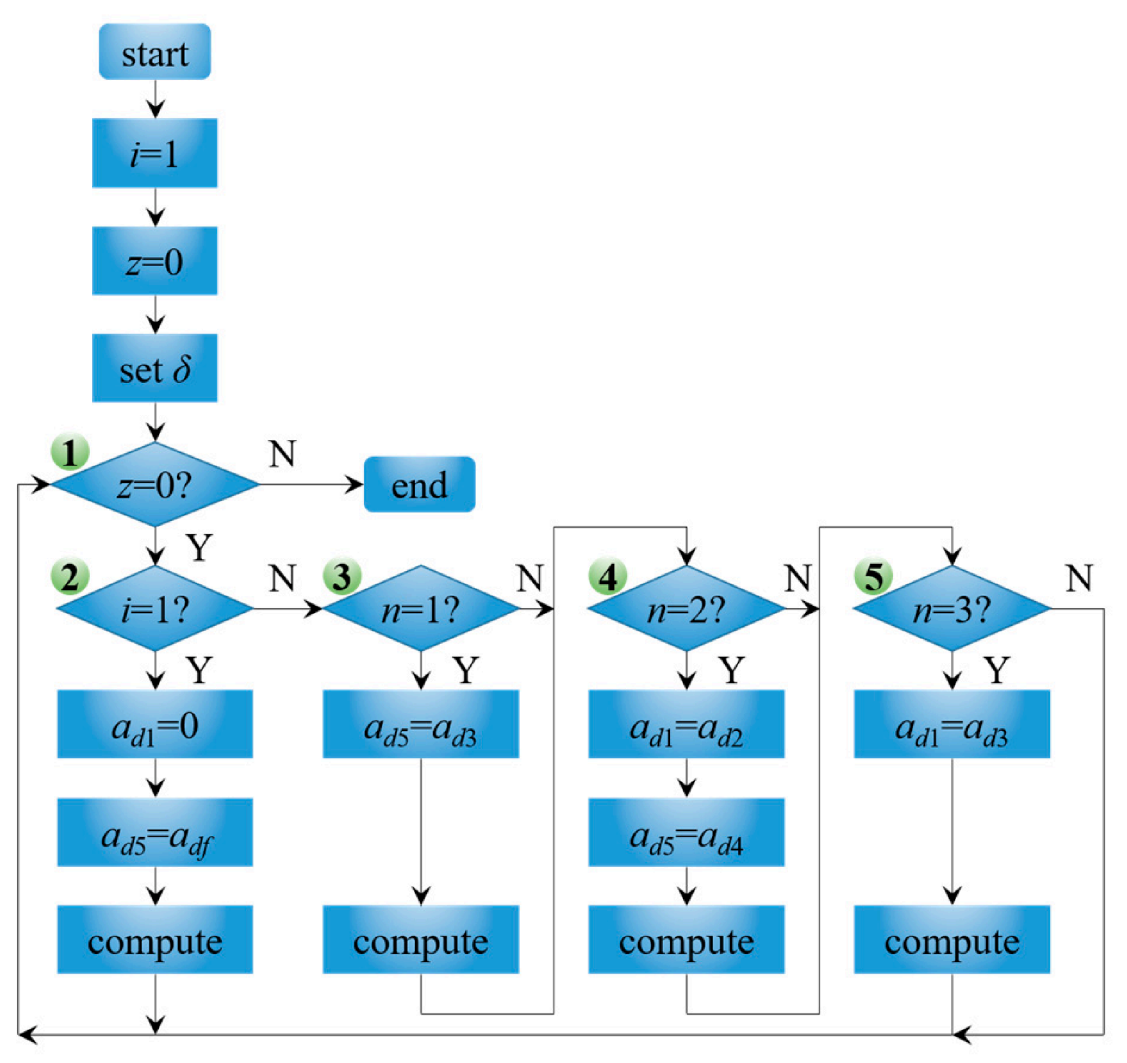
Computational structure of the current approach.

To overcome this shortcoming, this paper proposes an advanced numerical approach. Figure 3 shows the computational structure of this current approach. Specifically, three parameters are set: *i*, *z* and *δ*. The parameter *i* represents the calculation step. The parameter *z* represents the calculation flag. The parameter *δ* represents the relative error between the maximum BF obtained by the current approach and the traditional approach. Initially, the parameter *i* is set to one and the parameter *z* is set to zero.

After setting the input parameters *i*, *z* and *δ*, a main loop function is created. In this main loop function, the first judgement will be checked. In this first judgement, whether the parameter *z* equals zero will be checked. If the parameter *z* is not zero, this main loop function is terminated.

By contrast, if the parameter *z* is zero, the second judgement will be conducted. In this second judgement, whether the parameter *i* equals one will be checked. If the parameter *i* equals one, two intermediate parameters for the debonding region will be created: *a_d_*_1_ and *a_d_*_5_. Their value can be calculated with Equations (11) and (12).

*a_d_*_1_ = 0 (11)

*a_d_*_5_ = *a_df_* (12)

After that, the function of “compute” will be conducted. The computational structure of the “compute” function will be illustrated in Section 2.2. After the function of “compute” is conducted, the first judgement will be continuously checked.

For the second judgement, if the parameter *i* is not equal to one, the third judgement will be created. For this third judgement, whether the parameter *n* equals one will be checked. If the third judgement result is true, Equation (13) will be executed.

*a_d_*_5_ = *a_d_*_3_ (13)

After Equation (13) is executed, the function of “compute” will be executed. Then, the fourth judgement will be executed. The fourth judgement will also be executed if the result of the third judgement is false.

For this fourth judgement, whether the parameter *n* equals two will be checked. If the fourth judgement result is true, Equations (14) and (15) will be executed.

*a_d_*_1_ = *a_d_*_2_ (14)

*a_d_*_5_ = *a_d_*_4_ (15)

Then, the function of “compute” will be executed. Following this, the fifth judgement will be conducted. The fifth judgement will also be executed if the result of the fourth judgement is false.

For the fifth judgement, whether the parameter *n* equals three will be checked. If the judgement result is true, Equation (16) will be executed.

*a_d_*_5_ = *a_d_*_3_ (16)

Then, the function of “compute” will be executed. After that, the first judgement will be executed again. It should be noted that in the fifth judgement, if the parameter *n* is not equal to three, the first judgement will also be executed. When the first judgement result is false, the calculation is terminated.

### 2.2. Computational Structure of the “Compute” Function

**Figure 4:**
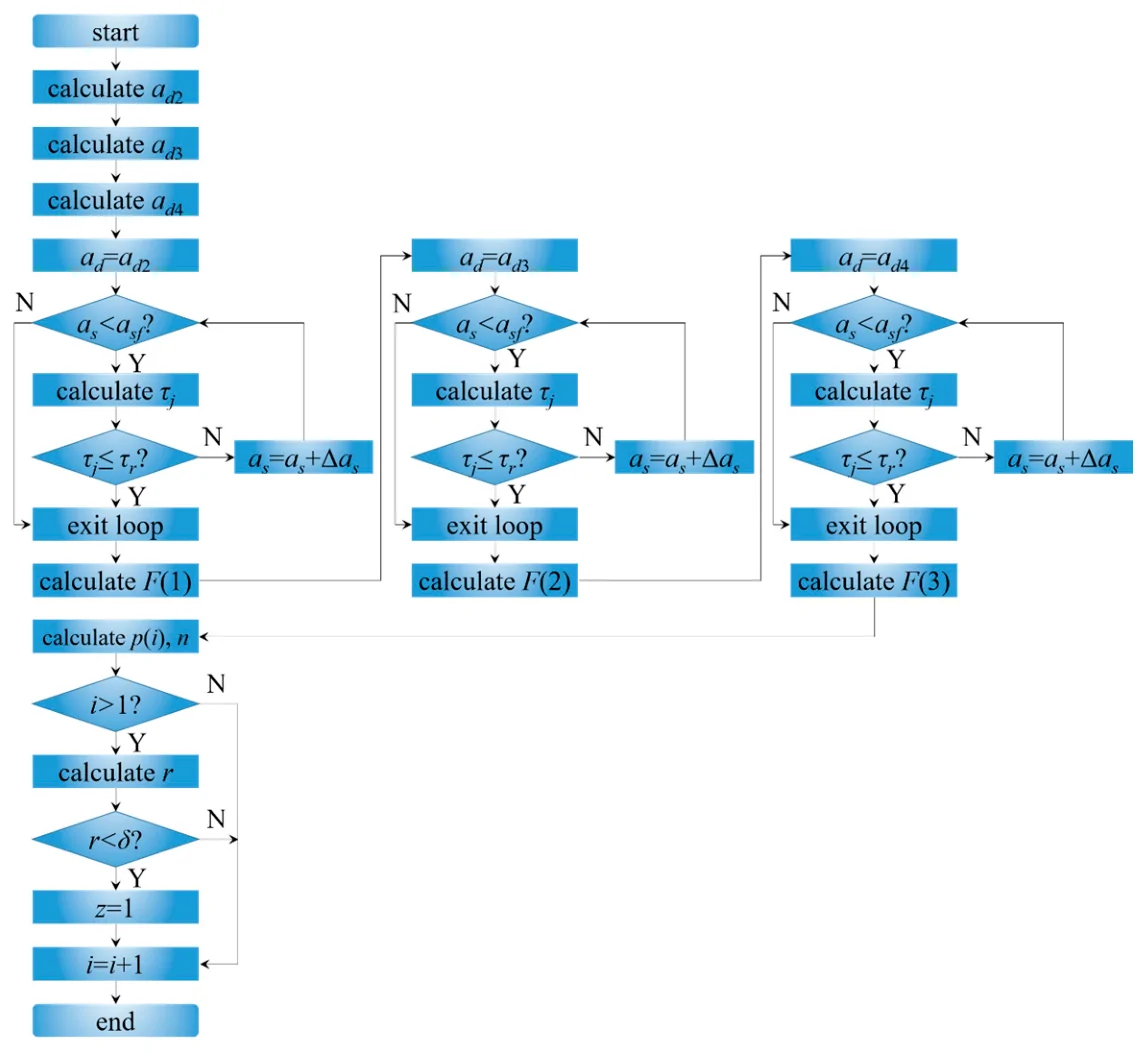
Computational structure of the function of “compute”.

In Section 2.1, a function of “compute” is established to calculate the proper length of the debonding region. For this function, the computational structure is drawn in Figure 4.

In this approach, five different debonding lengths will be calculated. The reason for using five different debonding lengths is that the proposed numerical approach is based on the bisection method. Therefore, for the debonding region, when the minimum length and the maximum length are determined, an intermediate length can be calculated. During calculation, to further reduce the range of the debonding region, another two positions are determined. One is located between the minimum length and intermediate length. The other is located between the intermediate length and maximum length.

For these five debonding lengths, the calculation procedures are listed below. First, the minimum debonding length can be calculated with Equation (11). The maximum debonding length can be calculated with Equation (12). After that, three parameters will be calculated, including *a_d_*_2_, *a_d_*_3_ and *a_d_*_4_. They can be calculated with Equations (17)–(19).

(17) $$a_{d2}=\frac{3a_{d1}+a_{d5}}{4}$$

(18) $$a_{d3}=\frac{a_{d1}+a_{d5}}{2}$$

(19) $$a_{d4}=\frac{a_{d3}+a_{d5}}{2}$$

where *a_d_*_2_, *a_d_*_3_ and *a_d_*_4_: three intermediate length parameters for the debonding region.

This numerical approach is fundamentally based on the bisection method. The purpose of using this bisection method is to continuously reduce the range of the debonding region.

After these three intermediate parameters for the debonding length (*a_d_*_2_, *a_d_*_3_ and *a_d_*_4_) are calculated, Equation (20) is executed.

*a_d_* = *a_d_*_2_ (20)

Then, a loop function is created to judge whether the parameter *a_s_* is smaller than the parameter *a_sf_*. If the judgement result is true, the parameter *τ_j_* will be calculated with Equation (21).

(21) $$\tau_{j}=\cos\left(\lambda_{2}a_{s}\right)-\frac{\lambda_{2}}{\lambda_{1}}\tanh\left(\lambda_{1}\left(L-a_{d}-a_{s}\right)\right)\sin\left(\lambda_{2}a_{s}\right)-\frac{\tau_{r}}{\tau_{p}}$$

where *τ_j_*: the shear stress at the point where the softening region ends.

If the parameter *τ_j_* is larger than the value of *τ_r_*, Equation (22) will be executed. The purpose of Equation (22) is to gradually increase the softening region until reaching its final length in the third course.

*a_s_* = *a_s_*+Δ*a_s_* (22)

where Δ*a_s_*: the incremental interval of the softening region.

When the softening length is larger than or equal to the value of *a_sf_*, this loop function will be terminated. Then, Equation (4) will be executed and the value will be assigned to the parameter *F*(1). Here, *F*(1) represents the BF for the specific debonding length of *a_d_*_1_.

Then, the parameter *a_d_*_3_ will be assigned to the parameter *a_d_*, as shown in Equation (23):

*a_d_* = *a_d_*_3_ (23)

After that, a loop function will be created to judge whether the parameter *a_s_* is smaller than the value of *a_sf_*. If the judgement result is true, Equation (21) will be executed to calculate the stress at the point where the softening region ends. Then, a loop function will be created to check whether the value of *τ_j_* is smaller than or equal to the value of *τ_r_*. If the judgement result is false, Equation (22) will be executed to get the latest softening length. When the latest softening length is larger than or equal to the value of *a_sf_*, this loop function will be terminated. Then, Equation (4) will be executed and the result will be assigned to the parameter *F*(2). Here, *F*(2) represents the BF for the specific debonding length of *a_d_*_3_.

Then, the parameter *a_d_*_4_ will be assigned to the parameter *a_d_*, as shown in Equation (24):

*a_d_* = *a_d_*_4_ (24)

After that, a loop function will be created to judge whether the parameter *a_s_* is smaller than the value of *a_sf_*. If the judgement result is true, Equation (21) will be executed to calculate the stress at the point where the softening region ends. Then, a loop function will be created to check whether the value of *τ_j_* is smaller than or equal to the value of *τ_r_*. If the judgement result is false, Equation (22) will be executed to get the latest softening length. When the latest softening length is larger than or equal to the value of *a_sf_*, the loop function will be terminated. After this loop function is terminated, Equation (4) will be executed and the value will be assigned to the parameter *F*(3). Here, *F*(3) represents the BF for the specific debonding length of *a_d_*_4_.

After the parameters *F*(1), *F*(2) and *F*(3) are obtained, the maximum value among these three parameters will be selected. Then, the selected result will be assigned to the parameter *p*(*i*). Meantime, the corresponding index will be assigned to the parameter *n*.

Then, a judgement is created to check whether the parameter *i* will be larger than one. If the judgement result is true, Equation (25) will be conducted. The purpose of Equation (25) is to judge whether the obtained maximum BF is the practical maximum BF. The convergence criterion is that when there is quite a small difference between the maximum BF in the current calculation loop and previous calculation loop, the obtained maximum BF can be accepted. Moreover, based on the bisection method and this judgement equation, this numerical approach can avoid the obtained result converging to a local maximum BF or overlooking the maximum BF.

(25) $$r=\frac{p\left(i\right)-p\left(i-1\right)}{p\left(i-1\right)}$$

where *r*: the relative difference between the maximum BF; *p*(*i*): the maximum BF in the current calculation loop; and *p*(*i* − 1): the maximum BF in the previous calculation loop.

Following this, the other judgement is created. Specifically, whether the parameter *r* is smaller than the parameter *δ* will be checked. When the parameter *δ* is small enough, the precision of the calculated maximum BF can be guaranteed. To confirm that the obtained maximum BF is stable and acceptable, this study recommends that the parameter *δ* can be set to 1%. It means that in the adjacent two calculation steps, the relative difference between the calculated maximum BF of the rock anchors is less than 1%. This can guarantee that the calculated maximum BF of the rock anchors is close to the exact solution.

If the judgement result is true, the parameter *z* will be set to one and Equation (26) will be executed. Then, the function of “compute” is terminated.

*i* = *i* + 1 (26)

It is noted that if the parameter *i* is smaller than or equal to one, Equation (26) is executed directly. Also, if the parameter *r* is larger than or equal to the parameter *δ*, Equation (26) is executed directly.

## 3. Experimental Validation

To confirm the effectiveness of the current approach, two experimental anchorage tests on rock anchors were adopted. The maximum BF obtained in the experimental anchorage tests is compared with the results obtained with the current approach. Moreover, to demonstrate the superiority of the current approach, the maximum BF obtained with the traditional approach is incorporated and compared.

### 3.1. Experimental Validation with the First Case

Zhu [23] conducted experimental anchorage tests on rock anchors. He indicated that for in situ cases, the rock anchors’ length may be quite long so that the rock anchors’ internal end can reach stable rock masses. Based on this concept, he conducted in situ anchorage tests on steel rock anchors in the city of Qingdao in China.

The tested anchor had a diameter of 28 mm and its yield strength was 340 MPa. The ultimate tensile strength of the tested anchors was 570 MPa. The whole length of the tested anchor was 8 m. The tested anchor was installed along the vertical direction. The bottom 6.5 m of the tested anchor was embedded in the drilled borehole. As for the top 1.5 m of the tested anchor, it was left free from the drilled borehole so that hydraulic pulling instruments could be installed.

Cement-based mortar of type M30 was poured into the drilled boreholes to bond the rock anchors with the surrounding rock. After this mortar fully cured, the anchorage test was conducted.

A hollow cylinder with a maximum capacity of 1000 kN and a maximum stroke of 300 mm was used to pull the rock anchor out from the surrounding rock. During testing, a force gauge of type MGH500 was used to record the anchorage force.

To simulate this experimental anchorage scenario, the current approach proposed in this study was used. When this current approach was used, there were nine input parameters. Basically, they can be divided into two categories. The first category of input parameters can be measured directly from the experimental anchorage tests. They were the rock anchor radius, rock anchor modulus, anchorage length of the rock anchors, surrounding rock modulus and surrounding rock cross-sectional area. In this case, the first category of input parameters is listed in Table 1.

As for the second category of input parameters, they should be calibrated from the experimental anchorage tests. They included the shear strength, shear slip at the shear strength, residual shear strength and shear slip at the residual shear strength. When calibrating these four parameters, the experimental force–displacement curve should be first analysed. At the initial loading stage, there was a linear relationship between the force and displacement. When this linear relationship ended, the corresponding displacement can be regarded as the shear slip at the shear strength. Meantime, the corresponding experimental BF can be substituted into Equation (27). Then, the shear strength can be obtained.

(27) $$F=\frac{2\pi r_{b}\tau_{p}\tanh\left(\lambda_{1}L\right)}{\lambda_{1}}$$

As for the left two parameters, they can be substituted into Equation (4). During the calibration process, when the overall force–displacement curve and the maximum BF agreed well with the experimental results, the calibration could be terminated. Based on these procedures, the second category of input parameters is listed in Table 2.

**Figure 5:**
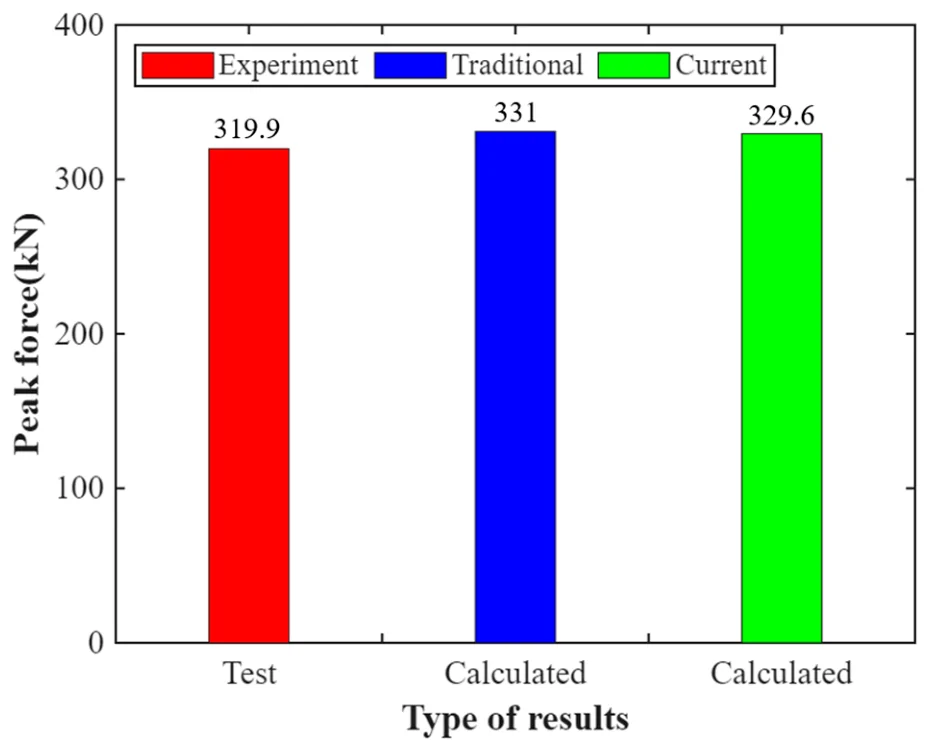
Comparison between the results obtained by the experiments, traditional approach and current approach.

Figure 5 compares the results obtained by the experimental tests and numerical simulation. In the experimental anchorage test, the maximum BF was 319.9 kN. With the current approach, the calculated maximum BF was 329.6 kN. The relative difference between them can be calculated with Equation (28):

(28) $$r_{F}=\frac{\left|F_{c}-F_{t}\right|}{F_{t}}$$

where *r_F_*: the relative difference between the BF obtained from the experimental anchorage tests and calculated with the current approach; *F_t_*: the BF obtained from the experimental anchorage tests; and *F_c_*: the BF calculated with the current approach.

Then, it was calculated that the relative difference between the maximum BF obtained from the experimental anchorage tests and calculated with the current approach was just 3%. This is a marginal relative difference. Therefore, it confirms the accuracy and credibility of the current approach.

It is noted that the maximum BF obtained by the traditional approach was also calculated, which was 331 kN. The relative difference between the experimental result and the result obtained with the traditional approach was 3.5%. Therefore, compared with the traditional approach, when the new approach is used, the relative difference between the experimental and calculated results is smaller.

To quantitatively analyse the correlation between the experimental force–displacement curve and the calculated force–displacement curve based on the current approach, the coefficient of determination is calculated with Equation (29).

(29) $$R^{2}=1-\frac{\sum_{i=1}^{n}{\left(F_{\mathrm{ti}}-F_{\mathrm{ci}}\right)}^{2}}{\sum_{i=1}^{n}{\left(F_{\mathrm{ti}}-F_{\mathrm{ta}}\right)}^{2}}$$

where *R*^2^: the coefficient of determination; *n*: the number of experimental displacement data; *F_ti_*: the *i*th experimental BF; *F_ci_*: the *i*th calculated BF based on the current approach; and *F_ta_*: the average experimental BF.

It should be noted that when calculating the coefficient of determination, the experimental BF and calculated BF at the same displacement were used. For the calculated BF, if the calculated displacement was not identical to the experimental displacement, the linear regression method was used to estimate the corresponding BF at the displacement that was equal to the experimental displacement. For this case, based on Equation (29), the calculated coefficient of determination was 95.7%. This indicates a satisfactory correlation between the experimental and calculated force–displacement curves.

**Figure 6:**
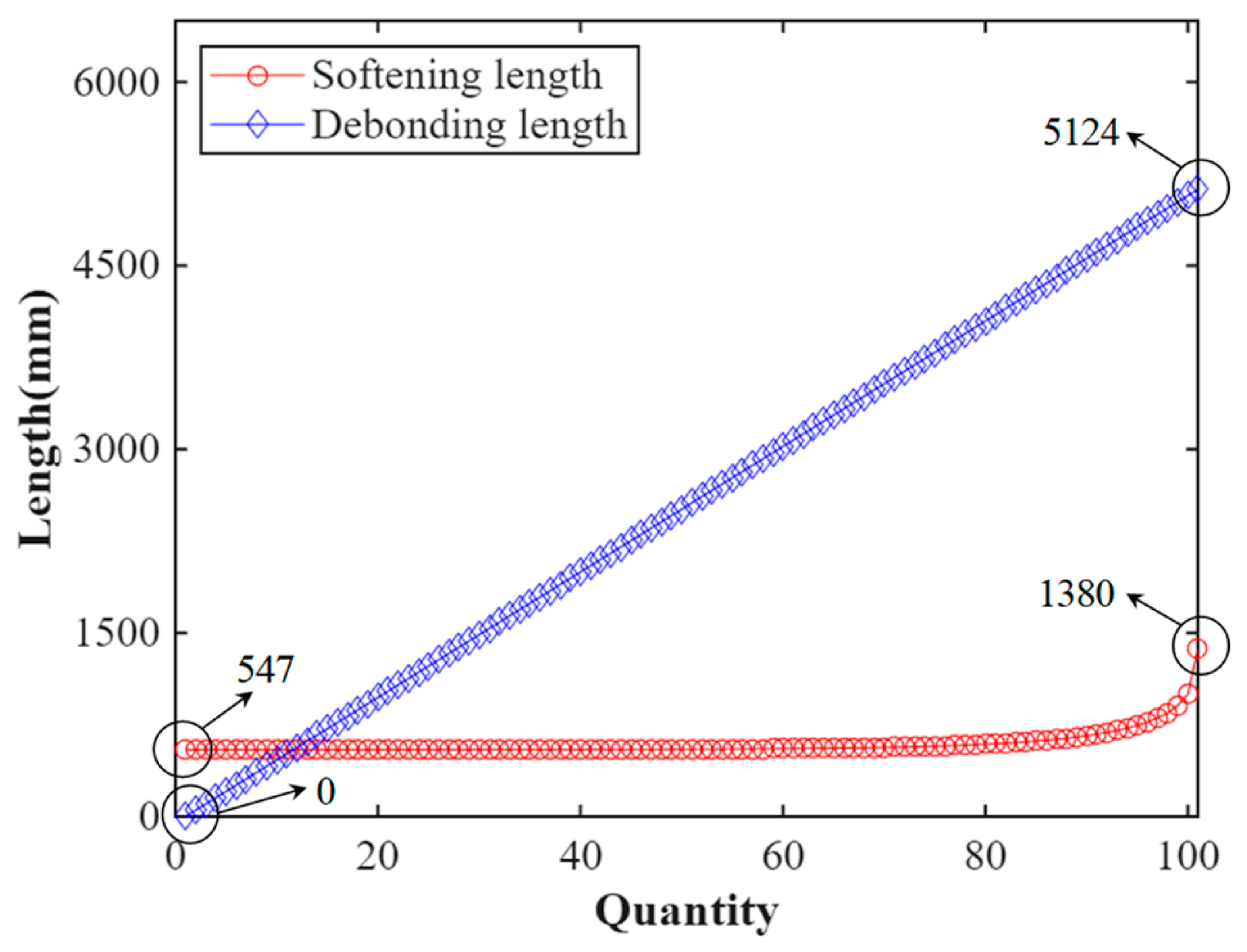
Variation process of the softening–debonding length in the third course.

Based on the traditional approach, the variation process of the softening–debonding region can be obtained. Specifically, in this experimental validation case, in the third course, the variation process of the softening–debonding region is shown in Figure 6. In this course, the debonding region’s length increased from zero to 5124 mm. Meantime, the softening region’s length increased non-linearly from 547 mm to 1380 mm. This variation process is consistent with previous research.

**Figure 7:**
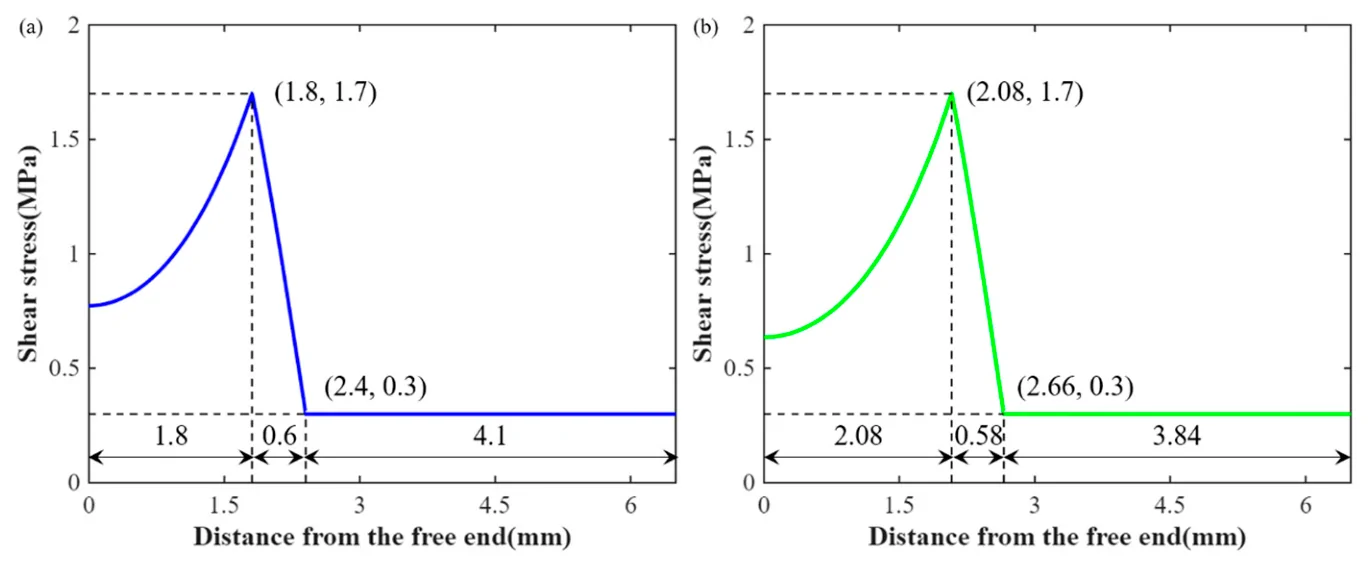
Stress distribution state along the interface obtained by numerical calculation: (**a**) the traditional approach; (**b**) the current approach.

In the third course, the stress distributed non-linearly along the interface. In the elastic region and softening region, the stress decreased non-linearly from the junction between these two regions towards two ends of rock anchors. As for the debonding region, the stress remained constant. In this experimental validation case, along the interface, the stress distribution state obtained by the traditional approach and current approach is shown in Figure 7.

There was generally a consistent correlation between the stress distribution obtained by the traditional approach and current approach. In the traditional approach, for the elastic region, the softening region and the debonding region, their lengths were 1.8 m, 0.6 m and 4.1 m. As for the current approach, their lengths were 2.08 m, 0.58 m and 3.84 m. For the softening length, the relative difference between the traditional approach and current approach was 3.3%. For the debonding length, the relative difference between the traditional and current approach was 6.3%. The above calculated results demonstrated that there was a marginal difference between the results obtained with the traditional approach and current approach. This further certified that the proposed approach was sufficient in calculating and predicting the anchorage performance.

**Figure 8:**
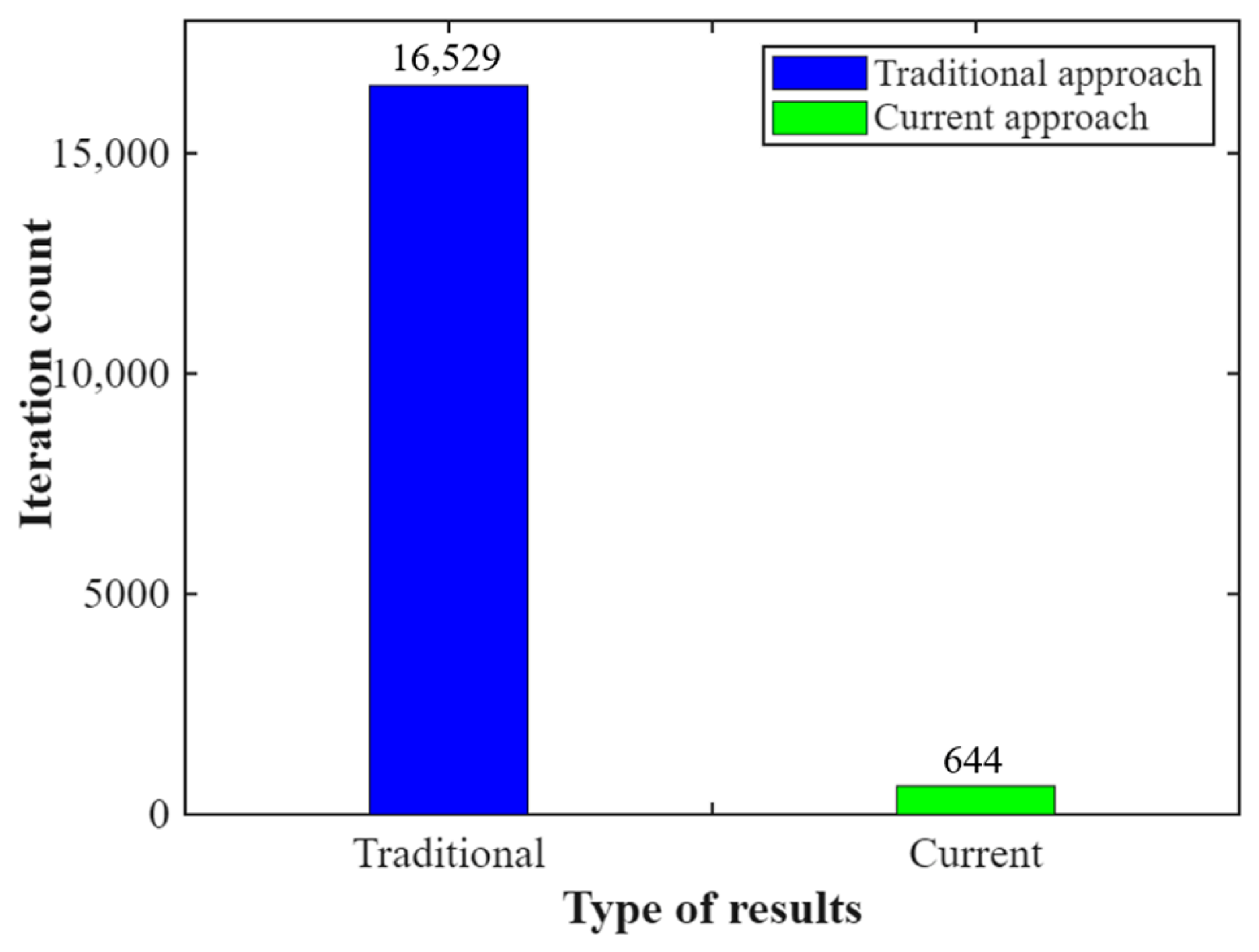
Comparison of the iteration count when two approaches were used in the first validation case.

The comparison of the computational efficiency between the traditional approach and current approach is shown in Figure 8. When the traditional and new approaches were used, the iteration count was 16,529 and 644. Therefore, compared with the traditional approach, when the current approach was used, the iteration count decreased by 96.1%. This indicates that compared with the traditional approach, the current approach showed apparent superiority.

When these two approaches were used, the calculation time was also counted. To realise this comparison, each approach was tested several times and the results were averaged. The results showed that when the traditional and new approaches were used, the calculation time was 5.01 ms and 3.32 ms. Therefore, when the new approach was used, less calculation time was needed.

### 3.2. Experimental Validation with the Second Case

To further validate the accuracy of the current approach, the experimental anchorage tests conducted by Ma [24] were used and compared. Specifically, Ma [24] conducted in situ anchorage tests on rock anchors. The tested anchor had a diameter of 32 mm and modulus of 204 GPa. The tested anchor was installed in pre-drilled boreholes. To bond the tested anchors with the surrounding rock, cement-based mortar of type C35 was used. A pumping pressure with a maximum value of 0.6 MPa was used to pump the grout into the boreholes. After pumping the cement-based mortar, the anchorage length was 10 m. The experimental test scenario used in this calculation is summarised in Table 3.

In this experimental anchorage test, a data logger system was used to record the BF. To numerically simulate this experimental anchorage test scenario, the parameters were calibrated and are listed in Table 4.

**Figure 9:**
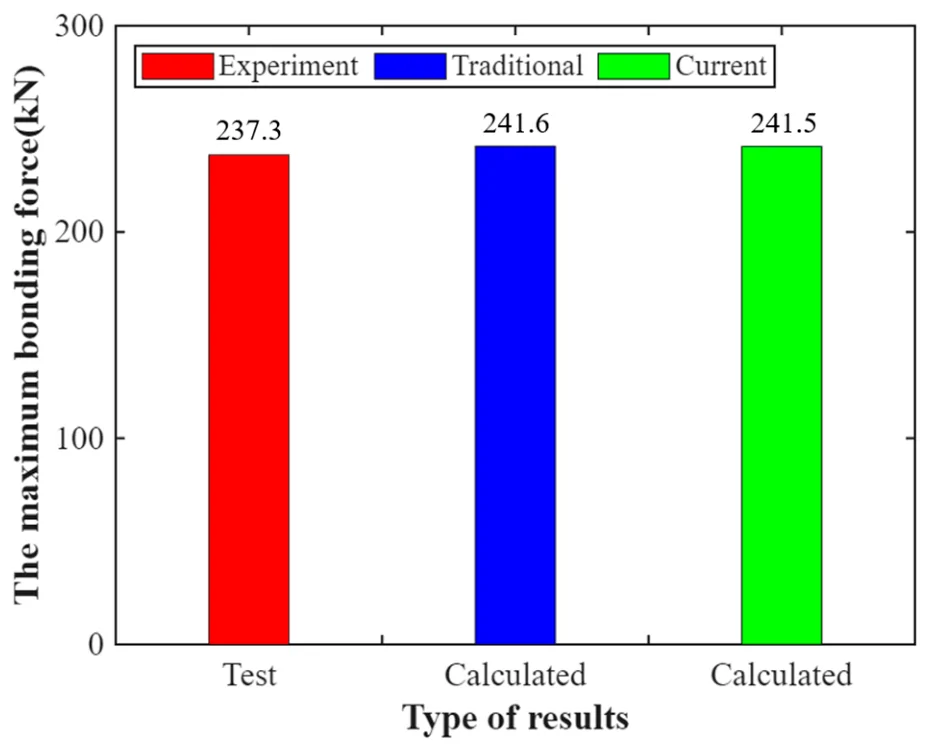
Comparison between the maximum BF obtained by the experiments, traditional approach and current approach.

The contrast between the maximum BF obtained in the experimental anchorage tests and the current approach is shown in Figure 9. In the experimental anchorage test, the maximum BF was 237.3 kN. Based on the current approach, the calculated maximum BF was 241.5 kN. Based on Equation (28), the relative difference between them was just 1.77%. This indicates quite a small difference between them.

As a comparison, the maximum BF obtained with the traditional approach was also given, which was 241.6 kN. The relative difference between the experimental result and it is 1.81%. This further confirms that compared with the traditional approach, when the new approach is used, the relative difference between the experimental and calculated results decreases.

As for the force–displacement curves obtained from the experimental anchorage tests and calculation based on the current approach, with Equation (29), the coefficient of determination was 99.8%. This indicates a high correlation between the experimental and calculated force–displacement curves based on the current approach.

**Figure 10:**
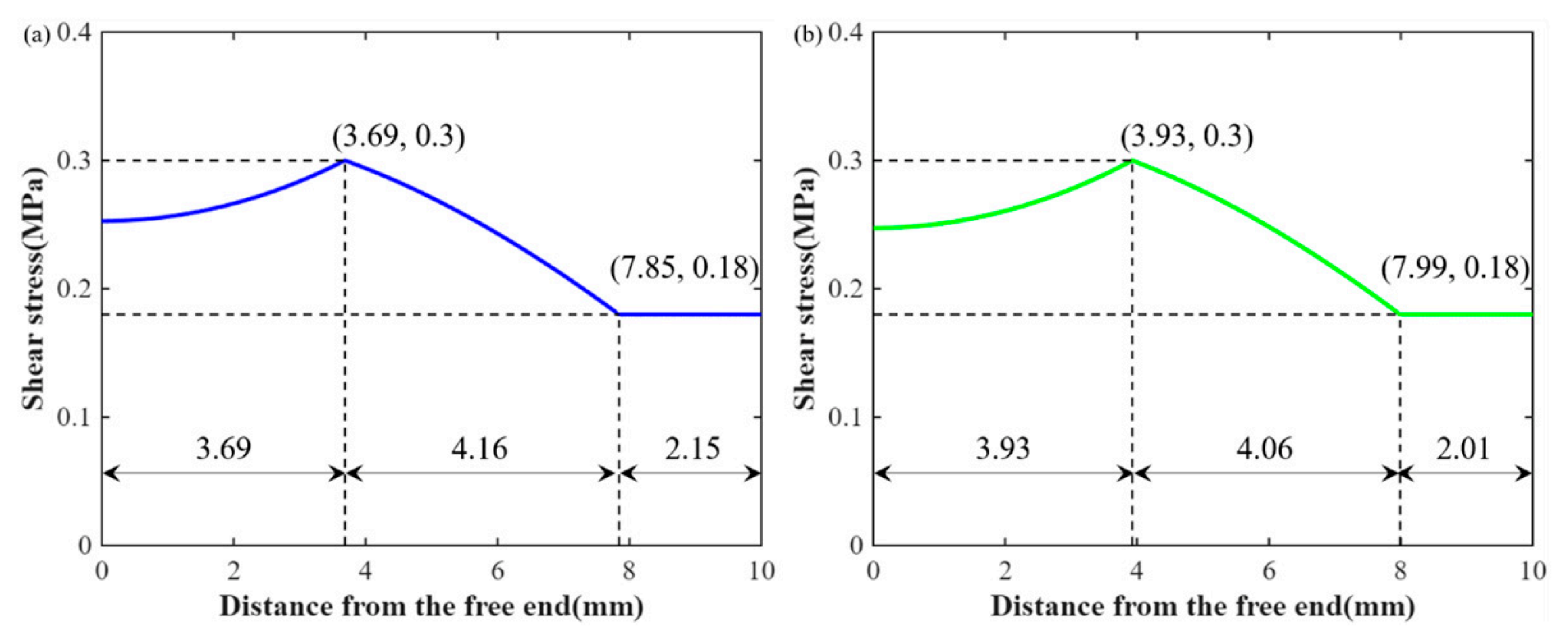
Stress distribution state along the interface calculated with two different numerical approaches: (**a**) the traditional approach; (**b**) the current approach.

When the maximum BF occurred, the stress distribution state was plotted (Figure 10). Apparently, when using two different numerical approaches, the stress distribution state was basically consistent.

When the traditional approach was used, for the elastic region, softening region and debonding region, their lengths were 3.69 m, 4.16 m and 2.15 m. By contrast, when the current approach was used, their lengths were 3.93 m, 4.06 m and 2.01 m.

Therefore, the relative difference between the elastic region length obtained from the traditional approach and current approach was 6.5%. The relative difference between the softening region length obtained from the traditional approach and current approach was 2.4%. The relative difference between the debonding region length obtained from the traditional approach and current approach was 6.5%. The above calculation demonstrated that there was not an apparent difference between the stress distribution state obtained from the traditional approach and current approach. This demonstrated that the current approach was sufficient in calculating the maximum BF and corresponding stress distribution state along the interface.

**Figure 11:**
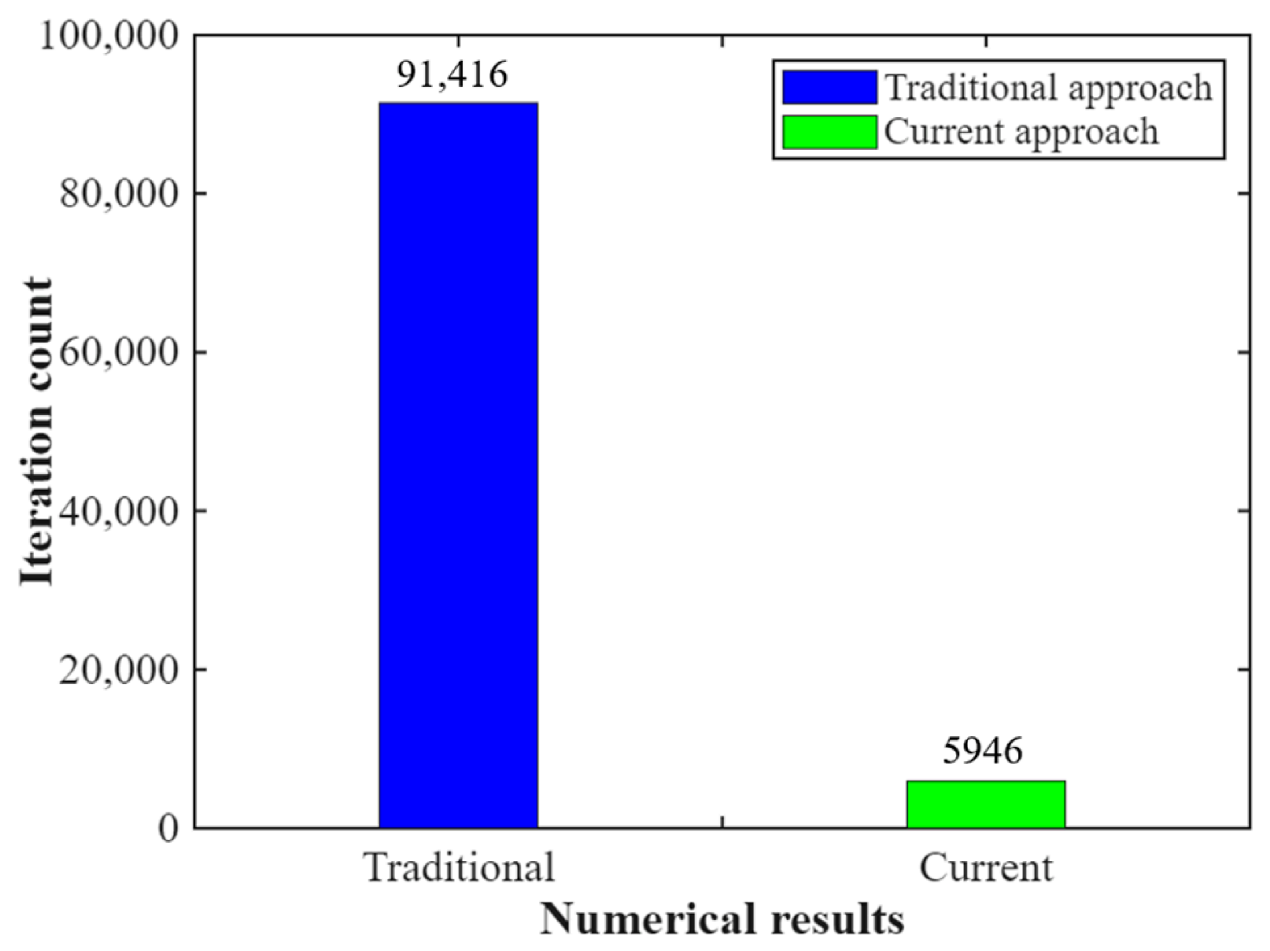
Comparison of the iteration count when two approaches were used in the second validation case.

Compared with the traditional approach, the current approach had better computational efficiency. When the traditional and new approaches were used, the iteration count was 91,416 and 5946 (Figure 11). It showed that after the new approach was used, the iteration count reduced by 93.5%. This further demonstrated the superiority of this current approach.

For the second case, the calculation time was also counted. The results showed that when the traditional and current approaches were used, the calculation time was 3.9 ms and 2.7 ms. This further indicates that when the current approach was used, less calculation time was needed.

The above two numerically calculated cases demonstrated that the current approach could get quite consistent results including the maximum BF and corresponding stress distribution state compared with the traditional approach. However, compared with the traditional approach, the computational efficiency of the current approach can be significantly improved.

## 4. Sensitive Analysis

To further understand the function and computational efficiency of the current approach, two sensitive analyses were conducted. In the first sensitive analysis, for the softening region, the number of elements was changed to indicate the computational efficiency of the current approach. In the second sensitive analysis, the grout shear strength was changed. The purpose was to reveal the influence of the grout shear strength on the anchorage performance and the computational efficiency of the current approach.

To conduct this sensitive analysis, a numerical anchorage test scenario was created. Specifically, rock anchors with a diameter of 22 mm and modulus of 210 GPa were tested. The anchorage length of this rock anchor was 2.4 m. The surrounding rock modulus was set to 12 GPa. Table 5 summarises the corresponding input parameters for this numerical anchorage test scenario.

As for the interface, its mechanical properties are tabulated in Table 6.

### 4.1. Computational Efficiency Comparison with Different Number of Elements

In the traditional and current approaches, the proper softening region length is obtained following the same computational structure. Specifically, for each debonding length, the softening region length increases from the initial value (*a_si_*) to the final value (*a_sf_*). To get the proper softening region, the domain ranging from the initial value to the final value is usually divided into a number of elements. For the softening region, when the number of elements changes, the corresponding iteration count will change.

**Figure 12:**
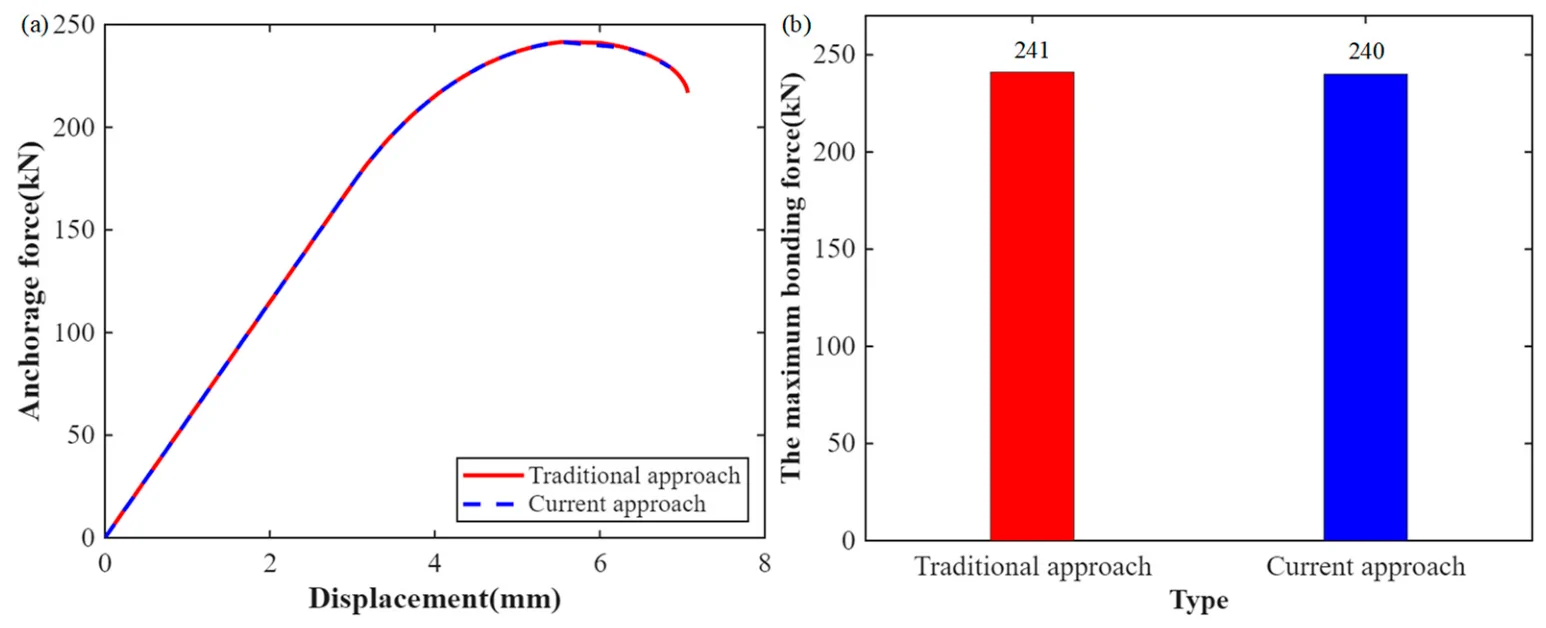
Comparison between the traditional approach and the current approach: (**a**) anchorage performance curves; (**b**) the maximum BF.

To evaluate the computational efficiency of this current approach, for the softening region, the number of elements increased from 500 to 3000. Then, the traditional and current approaches were adopted to conduct the calculation. The maximum BF was obtained (Figure 12). With the traditional approach, the maximum BF was 241 kN. With the current approach, the maximum BF was 240 kN. The relative difference between them was just 0.4%. This further confirmed that the current approach can properly calculate the maximum BF under different scenarios.

**Figure 13:**
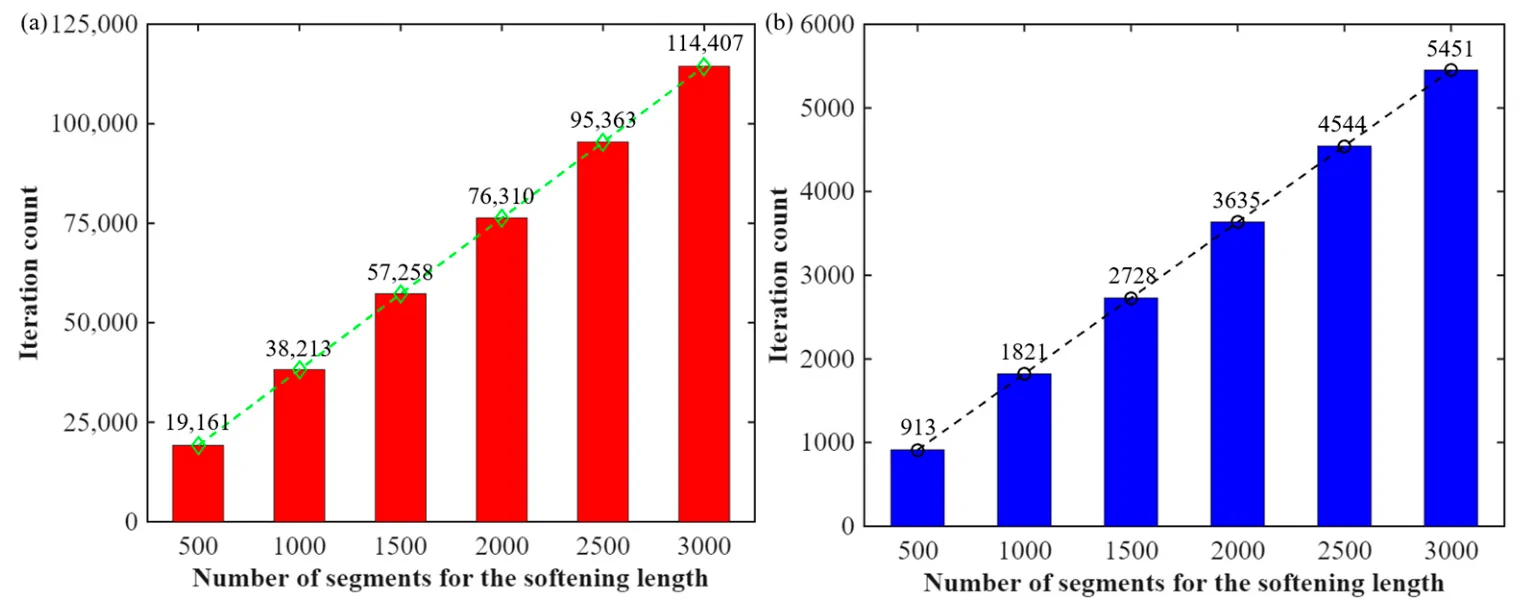
Comparison of the computational efficiency in the first sensitive analysis: (**a**) the traditional approach; (**b**) the current approach.

Meantime, the computational efficiency of the traditional approach and current approach was compared (Figure 13).

When the number of elements for the softening region increased from 500 to 3000, the iteration count increased from 19,161 to 114,407. It shows that when the traditional approach was used, a vast number of numerical calculations were needed. This apparently reduced the computational efficiency of the numerical calculation. By contrast, when the current approach was adopted, the iteration count significantly decreased. Specifically, for the softening region, when the number of elements increased from 500 to 3000, the iteration count increased from 913 to 5451. Compared with the traditional approach, when the current approach was used, the iteration count reduced by around 95.2%. This indicated that when the current approach was adopted, the computational efficiency could be significantly increased. This further demonstrated the superiority of the current approach.

### 4.2. The Influence of Grout Shear Strength on Anchorage Performance

**Figure 14:**
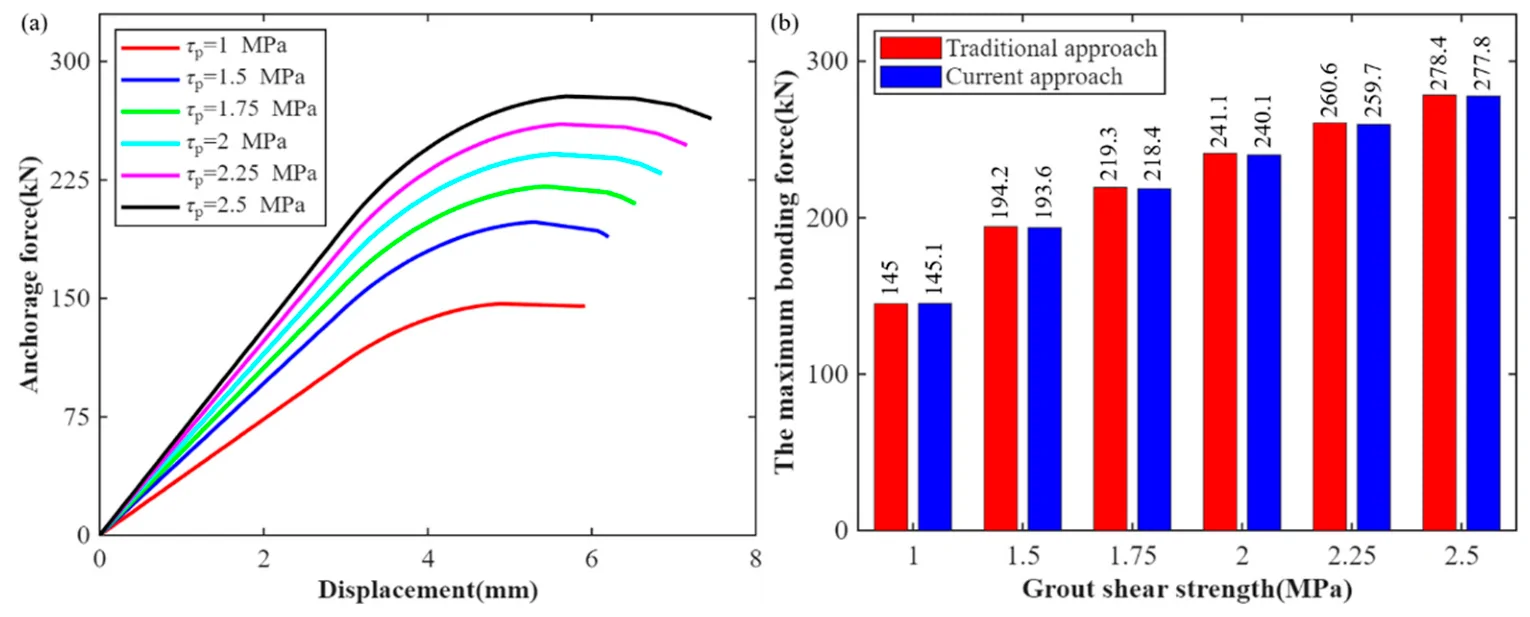
The influence of grout shear strength on anchorage performance: (**a**) anchorage performance curves obtained with the current approach; (**b**) the maximum BF obtained with two different approaches.

In the second sensitive analysis, the influence of the grout shear strength on the anchorage performance was studied. Specifically, the grout shear strength increased from 1 MPa to 2.5 MPa. Then, the maximum BF was calculated and obtained (Figure 14).

The results showed that when the grout shear strength increased from 1 MPa to 2.5 MPa, the maximum BF increased quickly. This was consistent with previous research [8]. This further confirmed the effectiveness of the current approach.

**Figure 15:**
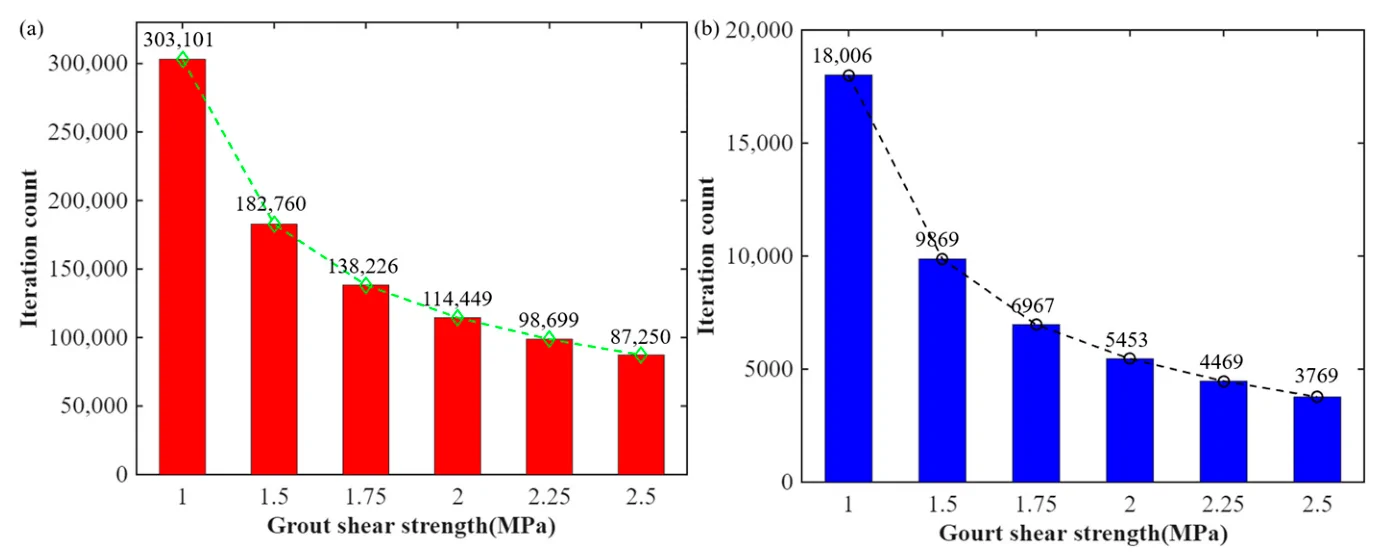
Comparison of the computational efficiency when the grout shear strength was different: (**a**) the traditional approach; (**b**) the current approach.

When the grout shear strength increased, the computational efficiency of the traditional approach and current approach was compared (Figure 15). When the grout shear strength increased, the iteration count decreased. However, the decreasing magnitude was different. For the traditional approach, the iteration count decreased from 303,101 to 87,250. By contrast, for the current approach, the iteration count decreased from 18,006 to 3769. Therefore, when the grout shear strength increased, compared with the traditional approach, the iteration count needed by the current approach reduced from 94.1% to 95.7%. This indicated that compared with the traditional approach, the current approach had significant superiority in calculation.

### 4.3. The Influence of Grout Shear Stiffness on Anchorage Performance

To evaluate the influence of the grout shear stiffness on the anchorage performance of rock anchors, this paper conducted a further calculation. The shear strength was set to 2.5 MPa. The shear slip at the shear strength varied from 1 mm to 3 mm. Based on Equation (30), the grout shear stiffness can be calculated, which varied from 0.83 MPa/mm to 2.5 MPa/mm.

(30) $$k_{g}=\frac{\tau_{p}}{\delta_{p}}$$

where *k_g_*: the grout shear stiffness.

**Figure 16:**
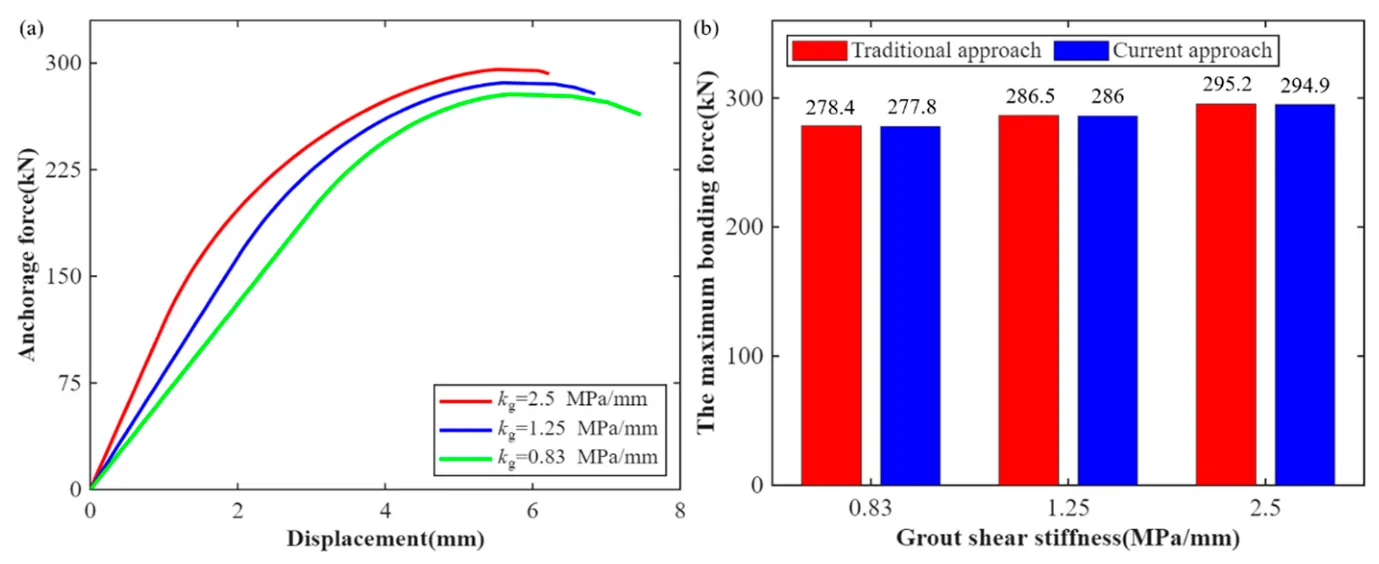
The influence of grout shear stiffness on anchorage performance: (**a**) anchorage performance curves obtained with the current approach; (**b**) the maximum BF obtained with two different approaches.

When the current approach was used, the calculated anchorage performance of the rock anchors is shown in Figure 16a. Apparently, when the grout shear stiffness increased, the stiffness of the whole rock anchorage system increased. Moreover, the maximum BF increased, which is depicted in Figure 16b. When the grout shear stiffness increased from 0.83 MPa/mm to 2.5 MPa/mm, the maximum BF of the rock anchors increased from 277.8 kN to 294.9 kN. Therefore, increasing the grout shear stiffness was beneficial for improving the stiffness and the maximum BF of the rock anchors. It was also noted that when the grout shear stiffness changed, the maximum BF obtained with the current approach was highly consistent with the maximum BF calculated with the traditional approach. This further indicated that compared with the traditional approach, the current approach can get consistent results with high efficiency.

Based on the results obtained in Section 4.2 and Section 4.3, the influencing degree of the grout shear strength and grout shear stiffness on the anchorage performance of the rock anchors was studied. The results showed that compared with the grout shear stiffness, the grout shear strength had a more significant influence on the maximum BF of the rock anchors. This can be demonstrated by the following data analysis. When the grout shear strength increased from 1 MPa to 2 MPa, increasing by 100%, the maximum BF increased from 145.1 kN to 240.1 kN, increasing by 65.5%. By contrast, when the grout shear stiffness increased from 1.25 MPa/mm to 2.5 MPa/mm, increasing by 100%, the maximum BF increased from 286 kN to 294.9 kN, only increasing by 3%.

## 5. Discussion

In this study, an advanced numerical approach was proposed to calculate the anchorage performance. Specifically, the maximum BF and the shear stress distributing at the interface were calculated. Two individual experimental anchorage test cases were adopted to demonstrate the credibility of the current approach. In these two experimental cases, the mechanical properties of the rock anchorage system and corresponding loading environment were different. Nevertheless, there was a consistent correlation between the experimental anchorage test results and calculated results. It indicates that this current approach can independently calculate the maximum BF of rock anchors.

The highlight of this current approach is that compared with the traditional approach, the computational efficiency can apparently be increased. Specifically, when this current approach is used, for each calculation loop, the whole debonding length is divided into five sections. Then, for these five debonding sections, the BF is calculated. Based on comparison, the maximum BF is obtained. Relying on this computational logic, the calculation loop was conducted. With this calculation loop, the domain of the debonding length can be quickly reduced. Then, within this reduced domain of the debonding length, the maximum BF can be obtained with high efficiency.

It is noted that when this new approach was used, the robustness of the computational efficiency was generally acceptable. In this study, different parameters were changed, including the discretised extent of the softening length and grout shear strength. For these different cases, when the new approach was used, the iteration count was consistently smaller than the traditional approach. This indicates that the superiority of the new approach was consistently reflected although the loading environment was different. However, it is admitted that the robustness of the new approach’s computational efficiency should be confirmed with further calculation cases.

The current study still has certain limitations. First, this study only focuses on the failure of the anchor/grout interface. In reality, failure of the grout/rock interface may also occur. Moreover, the failure of the grout/rock interface may have an influence on the maximum BF of rock anchors. However, at the current stage, the slip behaviour of the grout/rock interface is not considered. And the influence of the slip behaviour of the grout/rock interface on the maximum BF of rock anchors remains to be studied. Based on this limitation, a further theoretical study can be conducted to extend this study to other failure modes of the rock anchorage system.

Second, the credibility of this current approach is only demonstrated by two experimental cases. In these two experimental cases, the rock anchor type, grout properties and surrounding rock condition were different. Therefore, these two experimental cases demonstrate that this current approach can properly calculate the maximum BF of rock anchors when these conditions change. However, the shortcoming is that when these conditions change, how these parameters will influence the maximum BF of rock anchors has not been sufficiently evaluated and demonstrated. Therefore, in a further study, more experimental anchorage tests should be conducted to especially study the influence of the rock anchor type, grout properties and surrounding rock condition on the anchorage performance. Then, based on these series of experimental anchorage test results, the credibility of this current approach can be further demonstrated under different conditions.

Currently, more attention was paid to the debonding length. Moreover, the current approach was proposed to calculate and determine the proper debonding length when the maximum BF occurred. However, the limitation was that for each debonding length, the softening length was consistently varied from its initial value in the third course to its final value. Therefore, during the calculation, the domain of the debonding length was constant. In fact, there was a definite relationship between the softening region and the debonding region. When the debonding region changed, the domain of the softening region also changed. Moreover, the mobilised domain of the softening region was smaller than the original domain of the softening region. Then, for each debonding region length, if the mobilised domain of the softening region could be used, fewer iteration counts were needed. However, at the current course, no attempt has been conducted to determine the mobilised domain of the softening region.

Following this study, more research will be performed to analyse the variation process of the softening region for a specific debonding region. Moreover, an analytical approach or numerical approach should be proposed to determine the mobilised domain of the softening region. This is beneficial for further improving the computational efficiency of this current approach.

## 6. Conclusions

This paper proposed an advanced numerical approach to calculate the maximum BF. Meantime, when the maximum BF occurred, the corresponding length of the softening region and the debonding region could be obtained. Therefore, when the maximum BF occurred, the shear stress distribution along the interface could be plotted. The following conclusions were obtained.

(1) After the rock bolting system entered the third course, the debonding region was divided into five different values. The BF for these five different debonding lengths was calculated and compared. A computational loop was created. This computational loop was conducted until the maximum BF was obtained.

(2) Two independent experimental anchorage tests were adopted to confirm the accuracy of the current approach. The comparison results indicated that there was a close match between the maximum BF obtained from the experimental anchorage tests and the current approach. This confirmed that the current approach was sufficient in calculating the maximum BF.

(3) The computational efficiency of the traditional approach and current approach was compared. The results indicated that there was a marginal difference between the maximum BF obtained with the traditional approach and current approach. However, when the current approach was adopted, the iteration count could be significantly reduced. For the current approach, this computational superiority consistently occurred although the number of elements for the softening region varied.

(4) The grout shear strength apparently influenced the maximum BF. When the grout shear strength increased, the maximum BF increased. This indicated that in practical engineering, increasing the grout shear strength is beneficial for improving the overall bearing capacity of the rock reinforcement system. Although the grout shear strength was different, the computational efficiency of the current approach was consistently superior to the traditional approach.

## Figures and Tables

**Table 1:** Test scenario in the first experimental validation case.

*r_b_* (mm)	*E_b_* (GPa)	*L* (m)	*E_m_* (GPa)	*A_m_* (mm^2^)
14	200	6.5	5	1

**Table 2:** Calibrated parameters in the first experimental validation case.

*τ_p_* (MPa)	*δ_p_* (mm)	*τ_r_* (mm)	*δ_r_* (mm)
1.7	2	0.3	3

**Table 3:** Test scenario in the second experimental validation case.

*r_b_* (mm)	*E_b_* (GPa)	*L* (m)	*E_m_* (GPa)	*A_m_* (mm^2^)
16	204	10	10	1

**Table 4:** Calibrated parameters in the second experimental validation case.

*τ_p_* (MPa)	*δ_p_* (mm)	*τ_r_* (mm)	*δ_r_* (mm)
0.3	7	0.18	11

**Table 5:** Input parameters for the numerical anchorage test scenario.

*r_b_* (mm)	*E_b_* (GPa)	*L* (m)	*E_m_* (GPa)	*A_m_* (mm^2^)
11	210	2.4	12	1

**Table 6:** Input parameters in the sensitive analysis.

*τ_p_* (MPa)	*δ_p_* (mm)	*τ_r_* (mm)	*δ_r_* (mm)
2.5	3	0.5	6

## Data Availability

The original contributions presented in this study are included in the article. Further inquiries can be directed to the corresponding authors.
